# Context-based resolution of semantic conflicts in biological pathways

**DOI:** 10.1186/1472-6947-15-S1-S3

**Published:** 2015-05-20

**Authors:** Seyeol Yoon, Jinmyung Jung, Hasun Yu, Mijin Kwon, Sungji Choo, Kyunghyun Park, Dongjin Jang, Sangwoo Kim, Doheon Lee

**Affiliations:** 1 Department of Bio and Brain Engineering, KAIST, 291 Daehak-ro, Yuseong-gu, Daejeon 305-701, Republic of Korea; 2 Severance Biomedical Science Institute, Yonsei University College of Medicine, 50 Yonsei-ro, Seodaemun-gu, Seoul 120-752, Republic of Korea

## Abstract

**Background:**

Interactions between biological entities such as genes, proteins and metabolites, so called pathways, are key features to understand molecular mechanisms of life. As pathway information is being accumulated rapidly through various knowledge resources, there are growing interests in maintaining the integrity of the heterogeneous databases.

**Methods:**

Here, we defined conflict as a status where two contradictory pieces of evidence (i.e. 'A increases B' and 'A decreases B') coexist in a same pathway. This conflict damages unity so that inference of simulation on the integrated pathway network might be unreliable. We defined rule and rule group. A rule consists of interaction of two entities, meta-relation (increase or decrease), and contexts terms about tissue specificity or environmental conditions. The rules, which have the same interaction, are grouped into a rule group. If the rules don't have a unanimous meta-relation, the rule group and the rules are judged as being conflicting.

**Results:**

This analysis revealed that almost 20% of known interactions suffer from conflicting information and conflicting information occurred much more frequently in the literature than the public database.

With consideration for dual functions depending on context, we thought it might resolve conflict to consider context. We grouped rules, which have the same context terms as well as interaction. It's revealed that up to 86% of the conflicts could be resolved by considering context.

Subsequent analysis also showed that those contradictory records generally compete each other closely, but some information might be suspicious when their evidence levels are seriously imbalanced.

**Conclusions:**

By identifying and resolving the conflicts, we expect that pathway databases can be cleaned and used for better secondary analyses such as gene/protein annotation, network dynamics and qualitative/quantitative simulation.

## Background

The recent advent of molecular experiment and analysis technologies has led to an unprecedented success in discovering novel biological knowledge including biomedical entities and their interactions, or pathways. Pathways are at the center of modern biology by describing how biological molecules affect each other to emerge and manage life phenomena. Particularly, the entity-relation based model is the core of systems biology. Therefore, retrieving, collecting and integrating pathways relevant to a specific study constitute the most important first step.

Integrating pathway information is, however, not trivial. Most of currently known pathways data are only partially centralized for all the efforts to standardize syntax and ontology. Except a few cases, pathway data are physically scattered according to their biological domains. Some exist as a form of database, usually after confirming the reliability of the information by experts, or manual curation (MC) [1]. Databases provide a highly robust information source for researchers. However, their knowledge ranges are usually limited due to the laborious manual procedures. On the contrary, automated information extraction upon the millions of published studies, or literature mining (LM) covers ideally unlimited ranges of human knowledge [2]. In spite of the information quantity that LM provides, pathways recruited from LM can be suspicious due to several types of artifacts such as low literature quality and text mining errors. In this regards, integrating pathways from both MC and LM is required to compensate shortcomings from each resource.

Here we noticed an important but largely unexplored problem in the integrated biological pathways - a conflict. Information from different sources can be sometimes contradictory; for instance, one source may say a protein P activates a gene G's transcription, but at the same time, another source may say P represses G's transcription. This can be a desperate case for many researchers who rely on previously studied results. And we may want to ask several questions: 1) how much of the pathway information has conflict? 2) What causes conflicts? And 3) within a conflict, which one is more reliable? Stobbe et al. tried to answer a part of those questions by identifying consensus information among five different metabolic pathway databases [3]. Surprisingly, only 3% consensus was found among 6968 shared metabolic reactions.

**Figure 1:**
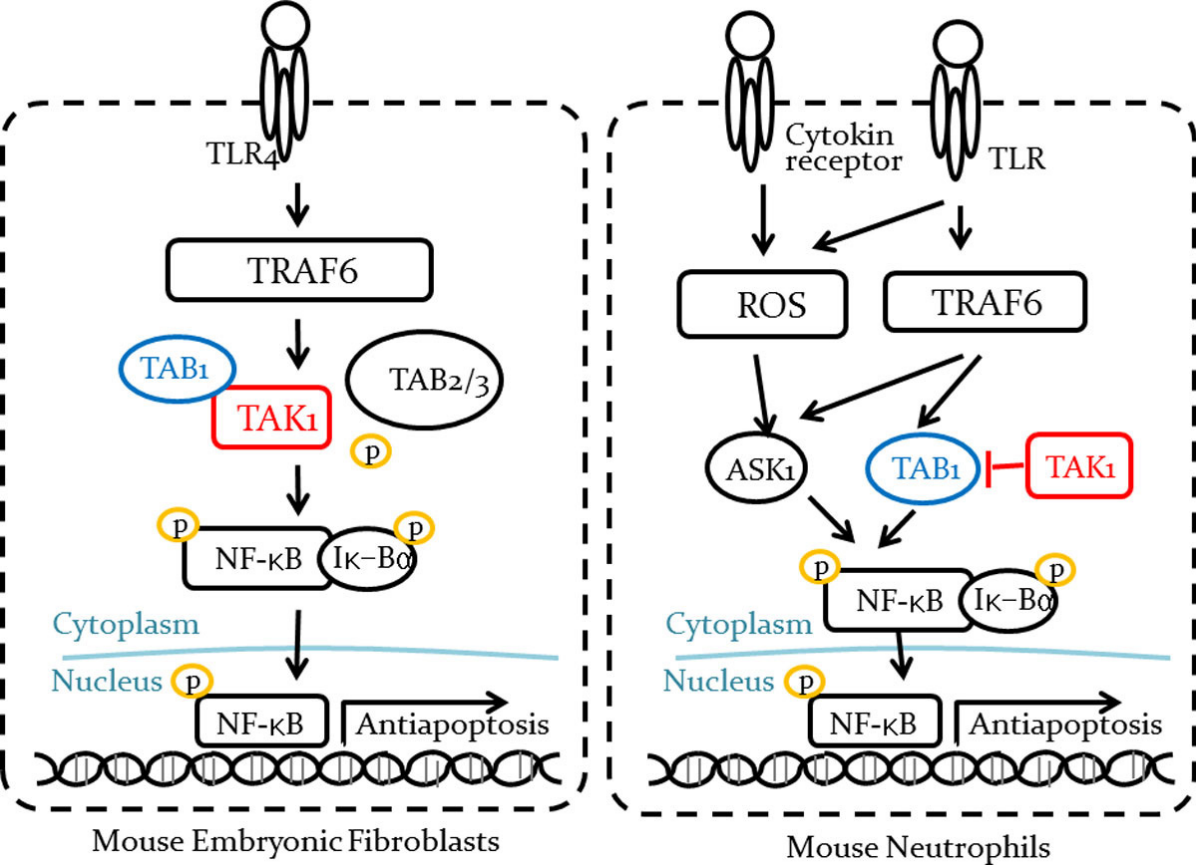
**Dual function of TAK1**. In mouse embryonic fibroblasts (MEFs), TAK1-TAB1 complex activates NF-κB signaling pathway to protect cells from apoptosis. However, in neutrophils, TAK1 binding prevents TAB1 from activating NF-κB signaling pathway, resulting in cell apoptosis.

We extended the concept of conflict to consider cellular context in the pathways. Context is basically an augment information set in a pathway; cell-type, organ, disease and drug. With a context, a pathway is more specified by the range where the context is true. For instance, "protein P activates gene G's transcription" can be specified like followings: "P activates G's transcription in brain", "P activates G's transcription in T-cells or "P activates G's transcription in schizophrenia". By considering contexts, we can prevent many false positive conflicts that are in fact non-contradictory pathways that do not overlap each other. Ajibade et al. discovered that TAK1-TAB1 complex works in a dual way [4]. TAK-TAB1 activates NF-κB signaling pathway in mouse embryonic fibroblasts; however, in neutrophils, TAK1 binding prevents TAB1 from activating NF-κB signaling pathway (Figure 1). It indicates that there may exist entities playing a role in some manner in one cell type but completely the opposite way in another cell type.

The context-dependent conflict analysis shows us how an integrated pathway dataset (from heterogeneous sources) should be handled. We found that an unexpectedly high portion of pathways (~20%) contain conflicting information. Thus, before continuing to the secondary analysis, conflicting rules must be taken care of. Cellular context information should be extracted at the highest level to distinguish false positive conflicts from true and specific conflicts. With a balance analysis, however, we also figured out that resolving conflicts by elimination (discarding one side of conflicts) is not trivial due to the tight competition between their evidence levels. But we could also check some obvious erroneous information from a part of them. We expect that subsequent studies on resolving conflicts, simulating over-conflicted pathways and measuring evidence level will dramatically improve the quality of our systems analysis.

## Methods

### Constructing an integrated biological pathway dataset

We first constructed a large-scale biological pathway set to interrogate the existence of conflicting information in our current knowledge. The pathway set consists of two major information resources for publicly reported biological pathways: a public canonical database and biomedical literatures.

#### Information extraction from public pathway database

We used the KEGG pathway database [5] as the test base of canonical pathways. Although the KEGG does not represent the entire pathway database, it provides highly reliable information through 281 manually confirmed human pathways that contain directional relationships between biomedical entities. Also, many associated tools and libraries enable a robust information extraction. At first, we extracted relation information from four canonical curated pathway databases including KEGG, PID, SMPDB, and ReconX. Among various types of conflict, we chose the conflict where contradictory information (i.e. 'A increases B' and 'A decreases B') exist in a same interaction. In case of 'react' or 'translocate' relation, it's not straight forward to choose which relations are contradictory with 'react' or 'translocate'. Most information in the databases except KEGG contains 'react' or 'translocate' information. In case of protein-protein interactions, the information is not used because they have no direction. We, therefore, only used the relation information in KEGG. Nonetheless, other pathway databases (i.e. BioCarta) can be added for additional analysis.

We downloaded KEGG Markup Language (KGML) files of 281 human pathways from KEGG pathway database using KEGGgraph package in R [6]. KGML files contain a set of records each of which consists of an entry field and a relation between two entities. We could further confirm the relational information using a KEGG ID (i.e. has:1432) in the entry field. Each relation has two entities, entity1 and entity2 where entry1 acts on entry2. Relations are annotated with interaction types (i.e. gene regulation, protein-protein interaction, and protein-chemical interaction) and subtypes (i.e. activation, inhibition, phosphorylation, dephosphorylation, glycosylation, ubiquitination, methylation, indirect effect, missing interaction, expression, repression, binding/association, dissociation, and reaction).

Based on the KGML file structure, we extracted 41,207 type/subtype-annotated relations among 5,457 biomedical entities.

#### Information extraction from biomedical literatures

Relations among biological entities were extracted from 13,214,710 PubMed abstracts. To accomplish this, we first tagged entities from the literatures using a set of existing Named Entity Recognition (NER) tools; different NER tools were used to tag different entity types.

We defined eight biomedical entity types including 'gene', 'protein', 'cell type', 'disease', 'organ', 'metabolite', 'drug' and 'biological process'. Based on the reported performance of NER tools using a manually curated corpus [7,8], we selected one best tool for each entity type. By this criterion, four NER tools were selected; Genia [9] for tagging 'gene', 'protein' and 'cell type', BANNER [10] for 'disease', LingPipe [11] for 'organ' and NERSuite [12] for 'metabolite', 'drug' and 'biological process'. According to the papers explaining the methods (see Table 1), we chose tools to extract each type of biological entities. The performances of GENIA was better than BANNER. Dataset which is used were, however, different. So we could not compare each other. These are the reason why we chose GENIA. The running time of GENIA was shorter than that of BANNER. And the coverage of GENIA was wider than that of BANNER, for example GENIA can find cell type in a literature.

**Table 1:** Performance of each tool.

Types	Tools	Date	**Prec**.	Recall	F-score	Choice	DataSet	Time*
**Chemical**	**CheNER**	2013	91%	30%	50%	O	EvalC Corpus	21 min
			
	**ChemSpot**	2012	75%	84%	79%	X		210 min

**Gene/** **Protein**	**GENIA tagger**	2006	66%	81%	72%	O	NLPBA2004 (Protein)	9 min

	**NERsuite**	2012	88%	82%	85%	X	BioCreative2	11 min
			
	**ABNER**	2005	68%	78%	73%	X		
			
	**BANNER**	2008	87%	83%	85%	X		60 min
			
	**Alias-i LingPipe** **(LIPI)**	2010	60%	70%	64%	X		

**Cell type**	**GENIA tagger**	2006	79%	71%	74%	O	NLPBA dataset	9 min
			
	**ABNER**	2005	80%	66%	72%	X		

**Disease**	**BANNER**	2008	87%	83%	85%	O	NCBI Disease Corpus	58 min

**Organ**	**Alias-i LingPipe** **(LIPI)**	2010				O	NCBI's GeneTag Corpus	16 min

Time* is time for which it takes to tag entities within 5,000 abstracts.

We considered that a sentence in a literature contains a relational information particularly when the relational word (i.e. increase, activate or repress) is located between two biomedical entities (see Table 2). For example, 'activate' is located between 'CYP1A' and 'benzo[a]pyrene' in following sentence; "In contrast, **CYP1A **isoforms can also **activate **some compounds, such as **benzo[a]pyrene **to their carcinogenic metabolite, and the induction of these isoforms increases the risk of carcinogenicity.".

**Table 2:** Pairs of entities in meta-relations

Meta-relation	Entity types acting roles	Target entity types
Increase	Gene/Protein	Gene/Protein
	
	Gene/Protein	Biological Process
	
	Gene/Protein	Drug
	
	Gene/Protein	Metabolite
	
	Gene/Protein	Disease
	
	Drug	Gene/Protein
	
	Drug	Biological Process
	
	Drug	Disease
	
	Disease	Gene/Protein
	
	Disease	Biological Process
	
	Disease	Metabolite
	
	Disease	Disease
	
	Biological Process	Gene/Protein
	
	Biological Process	Biological Process
	
	Biological Process	Metabolite
	
	Biological Process	Disease

Decrease	Gene/Protein	Gene/Protein
	
	Gene/Protein	Biological Process
	
	Gene/Protein	Metabolite
	
	Gene/Protein	Drug
	
	Gene/Protein	Disease
	
	Drug	Gene/Protein
	
	Drug	Biological Process
	
	Drug	Disease
	
	Disease	Gene/Protein
	
	Disease	Biological Process
	
	Disease	Metabolite
	
	Disease	Disease
	
	Biological Process	Gene/Protein
	
	Biological Process	Biological Process
	
	Biological Process	Metabolite
	
	Biological Process	Drug
	
	Biological Process	Disease

In this manner, totally 2,575,846 relations among 53,799 entities have been extracted from literature.

### Mapping relations to meta-relations

To investigate the consistency and inconsistency among interactions, we mapped the collected relationships between entities in the integrated pathways to two meta-relations, 'increase' and 'decrease'. As the word 'meta-relation' stands for, 'increase' and 'decrease' are not confined to their literal meanings, but include all the possible relations in which one entity affects the other in a positive or negative way. For instance, 'increase' may represent (but not be limited to) 1) increase of molecular quantity (i.e. activation of mRNA transcription), 2) activation of gene/protein functions (i.e. signal transduction by phosphorylation) and 3) induction of phenotypes (i.e. triggering of apoptosis or causing a disease). The 'decrease' meta-relation can be defined in a similar way.

We observed totally 14 relational terms from the KEGG database containing ['activation', 'inhibition', 'phosphorylation', 'dephosphorylation', 'glycosylation', 'ubiquitination', 'methylation', 'indirect effect', 'missing interaction', 'expression', 'repression', 'binding/association', 'dissociation', and 'reaction']. Among the 14, five relations including 'activation', 'phosphorylation', 'glycosylation' 'methylation' and 'expression' have been mapped to the 'increase' meta-relation. On the other hands, three relations including 'inhibition', 'dephosphorylation' and 'repression' have been mapped to the 'decrease' meta-relation. The remaining six terms could not be mapped due to their ambiguity in functional directionality.

In the biomedical literature pathways, we found 64 distinct relations (see Table 3). We mapped 36 relations to 'increase' and 28 relations to 'decrease'.

**Table 3:** Mapping table

Relations	Meta-relation
phosphorylate, glycosylate, acetylate, accelerate, accumul, activat, add, agoni, amplif, augment, elevat, encod, enhanc, enrich, express, generat, hyperexpr, improv, increas, increment, induc, mediat, overexpress, overproduc, produc, releas, result, secret, stimulat, synthesis, transactivat, transcri, translat, trigger, up-regulat, yield	Increase

attenuat, block, dephosphorylat, deacetylat, abolish, abrogat, antagoni, counteract, declin, decreas, degrad, deplet, depress, destruct, diminish, down-regulat, inactivat, inhibit, interfer, interrupt, obstruct, oppos, prevent, prohibit, reduc, remov, repress, suppress	Decrease

We considered the form of sentence to annotate the extracted meta-relations to their corresponding entities. When a sentence was written in an active form (i.e. "A increases B"), we regarded the former entity as the acting one and the latter as its target. On the contrary, we switched the roles of two entities when a sentence was written in a passive form (i.e. "A is increased by B"). We used a pattern relation extraction to derive the sentence form.

### Extracting context information

#### Definition of context

To identify relations that are valid under only a specific condition, we defined and considered four types of context: organ (OG), cell- type (CT), disease (DS) and drug (DR).

Organ and cell-type context provides the location where the corresponding relation arises. For instance, a statement 'A increases B in liver (OG)' constraints the relation's effect range to liver. Cell-type is similarly defined. Disease and drug context provides the clinical effect range of the corresponding relation with respect to phenotypic and environmental conditions respectively. For instance, 'A decreases B in lung adenocarcinoma (DS)' or 'A increases B in (or given) Herceptin' can be interpreted in a similar way.

To build the ontology of four different contexts, we obtained 1,191 organ, 526 cell-type and 4,620 disease terms from MeSH (Medical Subject Heading) database [13] (Table 4). For drug context, we used 6,825 terms from the DrugBank database [14]. Totally, 13,162 context terms were prepared for subsequent context information extraction.

**Table 4:** Statistics of four contexts and their resources

Context Class	Context Type	# Terms	Ref. DB
Locational	Organ (OG)	1,191	MeSH
		
	Cell Type (CT)	526	
		
Clinical	Disease (DS)	4,620	
	
	Drug (DR)	6,825	DrugBank

Total		13,162	

#### Extraction of context information

Based on the 13,162 context terms, we attempted to extract the context information associated to each relation in the constructed integrated biological pathway set. Given that the canonical pathway database does not contain any additional information other than entity-relation sets, the attachment of context information was only available for literature driven pathways.

We first searched for the defined context terms in the entire biomedical literatures. For the case that a context term *c *was discovered in a literature L, we annotated all the relations that have been extracted within L with *c*. When multiple context terms were found, we took the following approach. If the multiple terms consist of distinct context types (OG, CT, DS and/or DR), we annotated corresponding relations with the entire context set. If there are more than two context terms of a same type, we separated them into a set of single context to generate new relations, each of which is annotated with one context. In other words, context terms of different types are connected by an *AND*-like operation (i.e. 'A increases B in liver under Aspirin') whereas context terms of a same type are *OR*-like (i.e. 'A decreases B in liver or lung'). This strategy is consistent with the rationale that two same type contexts are not satisfiable simultaneously (i.e. there is no liver and stomach organ) or are rarely combined in a literature (i.e. 'stomach cancer and breast cancer' usually denotes two separated conditions).

### Identifying conflicting information

Based on the integrated biological pathway set with context information, we search for all conflicting (or contradictory) information that the set harbors.

#### Basic definitions

We define an *interaction ****i ***as a set of two entities and the associated meta-relation:

**i **= {LEFT-ENTITY, RIGHT-ENTITY, META-RELATION}

, where the *LEFT-ENTITY *is a biomedical entity (i.e. gene or protein) that plays an acting role in the relation, the *RIGHT-ENTITY i*s a target biomedical entity, and the *META-RELATION *is the relation between *LEFT-ENTITY *and *RIGHT-ENTITY *mapped to either of the previously defined 'increase' or 'decrease' relationship (see Methods 2.2). By this definition, one interaction corresponds to one edge in the context-free biological pathway.

We define a *rule ****r ***as a set of two entities, meta-relation between the entities and the associated context information:

***r ***= {*LEFT-ENTITY, RIGHT-ENTITIY, META-RELATION, CT, OG, DS, DR*}

, where *CT, OG, DS, DR *are sets of cell-type, organ, disease, drug context term(s) respectively. The context term fields can be '*null' *where no information was extracted. To exemplify the rule, two sample rules are denoted below, where ***r*_1 _**contains three context terms (CT, OG and DS) and ***r*_2 _**has no context information.

***r*_1 _**= {protein X, gene Y, increase, epithermal cell, small intestine, T2D, '*null'*}.

***r***_2 _= {protein A, gene B, decrease, '*null'*, '*null'*, '*null'*, '*null'*}

By definition, each interaction *has *a set of supporting rules (at least one rule). We define a *rule group *of an interaction ***i***, ***R***(***i***) as the set of rules the share the same *LEFT-ENTITY *and *RIGHT-ENTITY*:

***R***(***i***) = {***r ***| ***r***[1]=***i***[1], ***r***[2]=***i***[2]}

, where ***r***[1], ***r***[2], ***i***[1], ***i***[2] denotes the *LEFT-ENTITY *and the *RIGHT-ENTITY *of the rule ***r ***and the interaction ***i ***(as the way of referring an element by the address in the set). The rule group is further divided into two sub groups by the directionality of meta-relation. An *increase rule group ****R***(***i***)^+ ^and a *decrease rule group ****R***(***i***)^- ^of an interaction ***i ***are defined as:

***R***(***i***)^+ ^= {***r ***| ***r***[1]=***i***[1], ***r***[2]=***i***[2], r[3]='increase'}

***R***(***i***)^- ^= {***r ***| ***r***[1]=***i***[1], ***r***[2]=***i***[2], r[3]='decrease'}

, where ***r***[3] is the *META-RELATION *of the rule ***r***.

#### Judgment of conflict

For an interaction ***i ***and its supporting rule group ***R***(***i***), we generally expect the entire set ***R***(***i***) to be uni-directed, which means the observed relations between two entities should be consistent. Likewise, the coexistence of contradictory information (i.e. 'A increases B' and 'A decreases B') is exceptional and needs to be resolved for a proper downstream pathway analysis.

We call an interaction ***i ***has *conflicting information *where both of increase and decrease rules are contained in its supporting rule group. Using the previous definition, this condition can be defined as:

N(***R***(***i***)^+^) > 0 and N(***R***(***i***)^-^) > 0.

The *number of conflicts *in ***i ***is calculated as the sum of the number of rules, N(***R***(***i***)^+^) + N(***R***(***i***)^-^).

#### Judgment of context-dependent conflict

Using the context information, the effect range of a relation could be constrained (see Methods 2.3). Thus, a conflict between two rules that have non-overlapping effect ranges can be resolved. For instance, a rule 'A increases B in liver' and 'A decreases B in adipocyte' can be non-contradictory. Based on the annotated context, we further extend the definition of conflicting information. So, a *context-dependent conflict *arises only between rules that have identical context information. Formally, *a rule subgroup *of an interaction ***i***, ***R***(***i***, *OG, CT, DS, DR*) is defined as a subset of ***R***(***i***) that have the corresponding context.

***R***(***i**, OG, CT, DS, DR*) = {***r ***| ***r***[1]=***i***[1], ***r***[2]=***i***[2], ***r***[4]=*CT*, ***r***[5]=*OG*, ***r***[6]=*DS*, ***r***[7]=*DR*}

The possible number of subgroups is the number of combinations among four contexts.

Similarly, an *increase rule subgroup *and a *decrease rule subgroup *can be defined:

***R***(***i**, OG, CT, DS, DR*)^+ ^= {***r ***| ***r***[1]=***i***[1], ***r***[2]=***i***[2], ***r***[3]='increase', ***r***[4]=*CT*, ***r***[5]=*OG*, ***r***[6]=*DS*, ***r***[7]=*DR*}

***R***(***i**, OG, CT, DS, DR*)^- ^= {***r ***| ***r***[1]=***i***[1], ***r***[2]=***i***[2], ***r***[3]='decrease', ***r***[4]=*CT*, ***r***[5]=*OG*, ***r***[6]=*DS*, ***r***[7]=*DR*}

Therefore, context-dependent conflict arises when an *increase rule subgroup *and the corresponding *decrease rule subgroup *are both non-empty.

### Balance analysis among conflicting rules

We call two rule groups ***R***(***i***)^+ ^and ***R***(***i***)^- ^or two rule subgroups ***R***(***i**, OG, CT, DS, DR*)^+ ^and ***R***(***i**, OG, CT, DS, DR*)^- ^are *competitive *if they conflict. A balance relationship between two conflicting rule groups can be inferred from the number of supporting rules. We call a rule group is *strong *if the number of the group is bigger than that of its competing group, and *weak *otherwise.

To see if a conflict is dominated by one rule group, we define a *minor rule frequency MRF *as the size ratio of weak rule group over the whole rule groups:

*MRF *= ***min***(N(***R***(***i***)^+^), N(***R***(***i***)^-^)) / (N(***R***(***i***)^+^) + N(***R***(***i***)^-^))

, where ***min***(A, B) is the smaller number between A and B. An *MRF *near 0.5 denotes that two rule groups are supported in a similar level. A very small *MRF *may implicit the weak rule group resulted from erroneous literature information or mining procedures. We call a conflict is *dominating *if MRF < 0.25 and *balanced *otherwise.

## Results

### Integrated pathway networks

**Figure 2:**
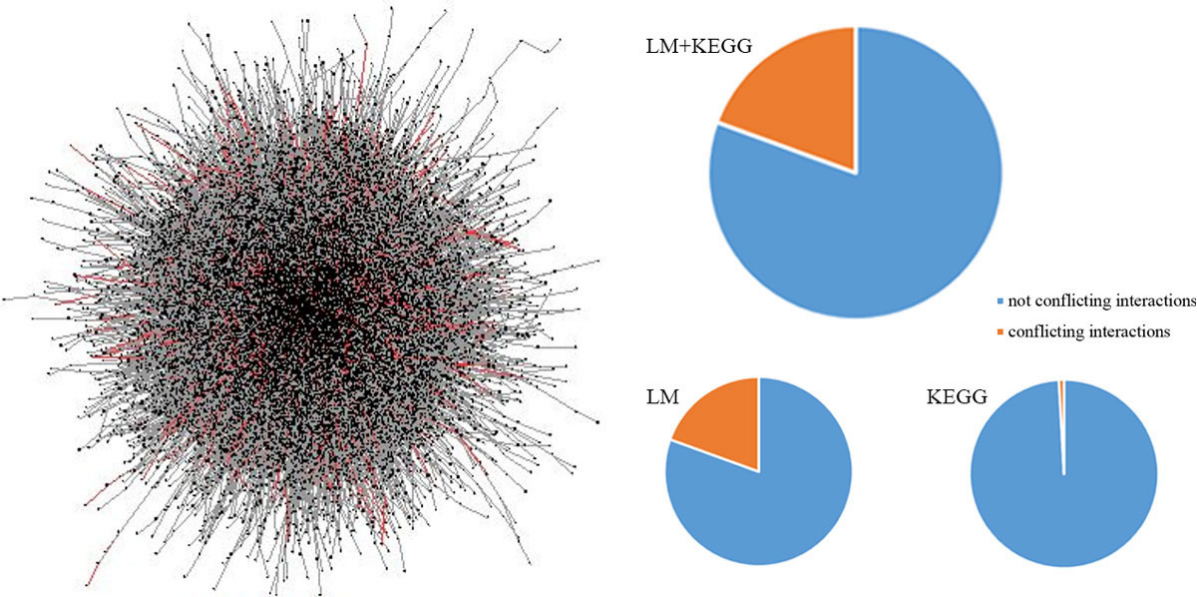
**Overall shape of a partial integrated pathway and ratios of conflicting interaction**.

We constructed the integrated pathway networks consist of 810,073 interactions (edges) among 53,799 biomedical entities from a canonical curated pathway database (KEGG database) and literatures. The 810,073 edges are comprised of 2,575,846 *rules *(see Methods). To show the overall shape of the integrated pathway network, we visualized a part of the whole network (about 5%) using Cytoscape [15] (Figure 2 left); drawing the entire network was computationally intractable. The average number of neighbors in the network was 4.5 and the characteristic path length was 4.7.

Among rules which are collected by parsing KEGG database, it's showed that 89% of interaction of them are also found in the interactions extracted by literature mining. This indirectly validated the accuracy of the extraction process.

### Conflicting information in integrated pathway networks

We found 157,065 out of 810,073 interactions (19.4%) contain conflicting information. The 157,065 conflicting interactions were supported by 1,564,315 rules (out of 2,575,846 rules, 60.7%). The ratio of conflicting interaction was unexpectedly high, showing that the collection of biological interactions suffers from self-inconsistency. The overall distribution of conflicts is depicted as red edges in Figure 2.

We found the ratio of conflicting interactions is dependent on the pathway resources. When using KEGG only, the ratio was extremely small 310 out of 35,845 (0.86%) to imply that most of the conflicting information came from literature mining. This is consistent with our expectation that pathways from more reliable sources contain conflicting information in a lower level.

### Analysis of context-dependent conflicts

As we described in Methods, a conflict between two entities can be resolved by constraining the effect range of the supporting rules. We applied four types of contexts, cell-type (CT), organ (OG), disease (DS), and drug (DR), to analyze the portion of the context-dependent conflicts.

To identify the effect of each context type, we applied the combination of the four contexts (16 cases) to the pre-identified conflicts. When applying each combination of context ***C ***(***C ***is a subset of {*CT, OG, DS, DR*}), we took the following approach. First, we generated a rule subgroup (see Methods) ***R***(***i***, ***C***) with respect to the context combination from the rule group of an interaction ***i***, ***R***(***i***). Second, we classified the rule subgroup into an increase rule subgroup ***R***(***i***, ***C***)^+ ^and ***R***(***i***, ***C***)^-^. Third, we counted the rules in the rule subgroup for which both increase and decrease rule subgroups are non-empty.

**Figure 3:**
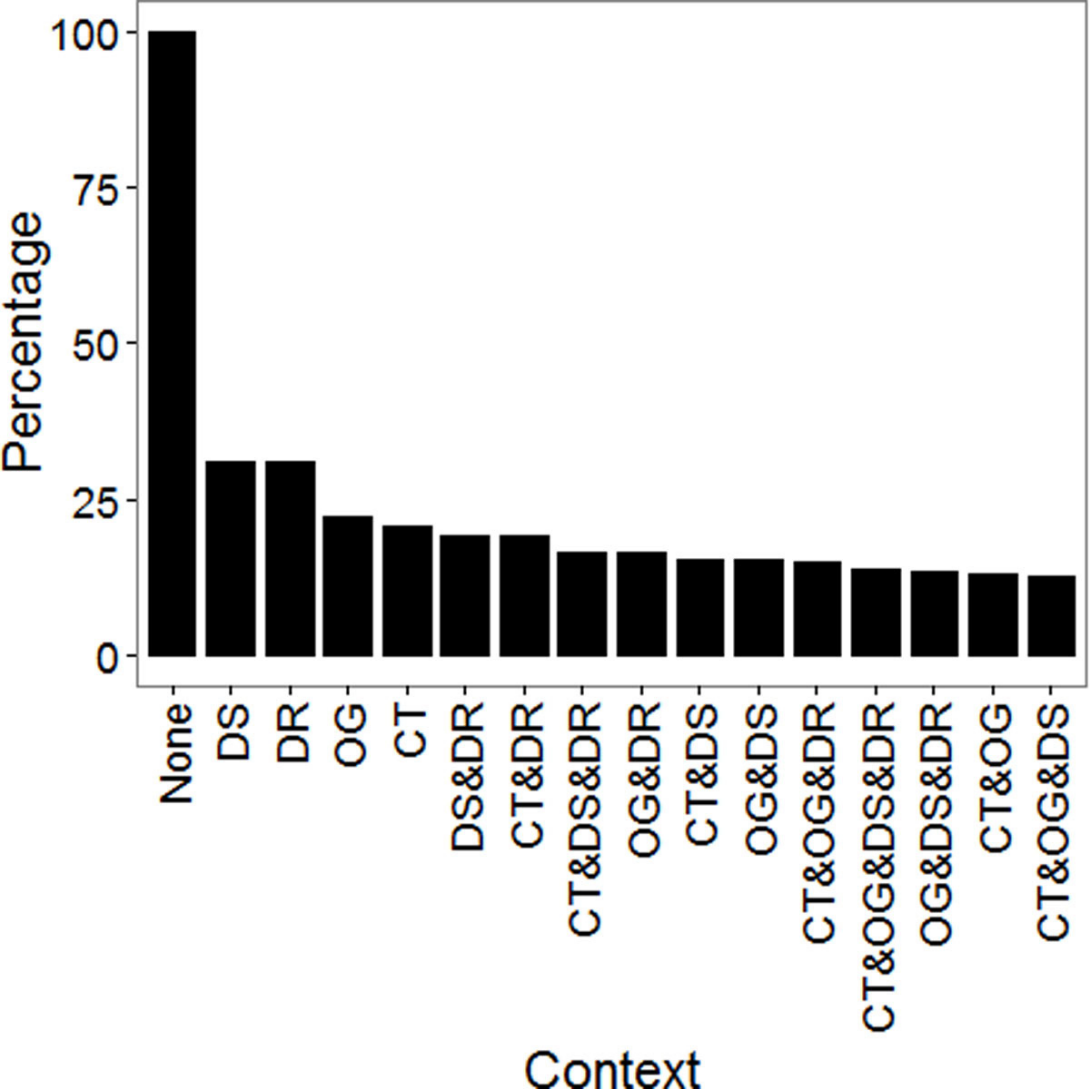
**Percentage of conflicting R(i, C)s**.

We found the ratio of conflicting rule groups have been dramatically reduced up to 13.6 % when considering contexts (Figure 3). Intuitively, application of more number of contexts resolves more conflicts. The result is consistent with the intuition; however, the effect of single context was significant too.

In general, we found the effect of locational contexts (organ and cell-type) is stronger than that of clinical context (disease and drug). This may imply that molecular functions and interactions in a cell vary more widely depending on the organs and tissue types. A well-known explanation of this is a tissue-specific regulation. Yu et al. identified 6,232 putative cis-regulatory modules regulating 2,130 tissue specific genes [16]. On the other hands, drugs are likely to change gene regulation and molecular activities in a more narrow way to target a specific function.

### Balance analysis of competing rules

Conflicts can be seen as a competition between two contradictory rule groups (increase versus decrease). There are many possible causes of the conflicts. One is that one rule group is supported by unreliable information. Some of the pathway sources contain erroneous information or there could be erroneous procedures in information curation and extraction. Some supporting literatures contain false discoveries, old hypothesis or discarded biological knowledge. The other possibility is that both rules are not wrong. Genes may function in a dual way or their functions are differed by unconsidered contexts.

Balance analysis is to see if one group is dominantly supporting than the other. As described in Methods, a minor rule frequency (MRF) can work as an indicator of the balance. A severe imbalance may imply one rule group is wrong. By definition, we call a conflict is a *dominating *conflict when MRF is less than 0.25, and *balanced *otherwise.

**Figure 4:**
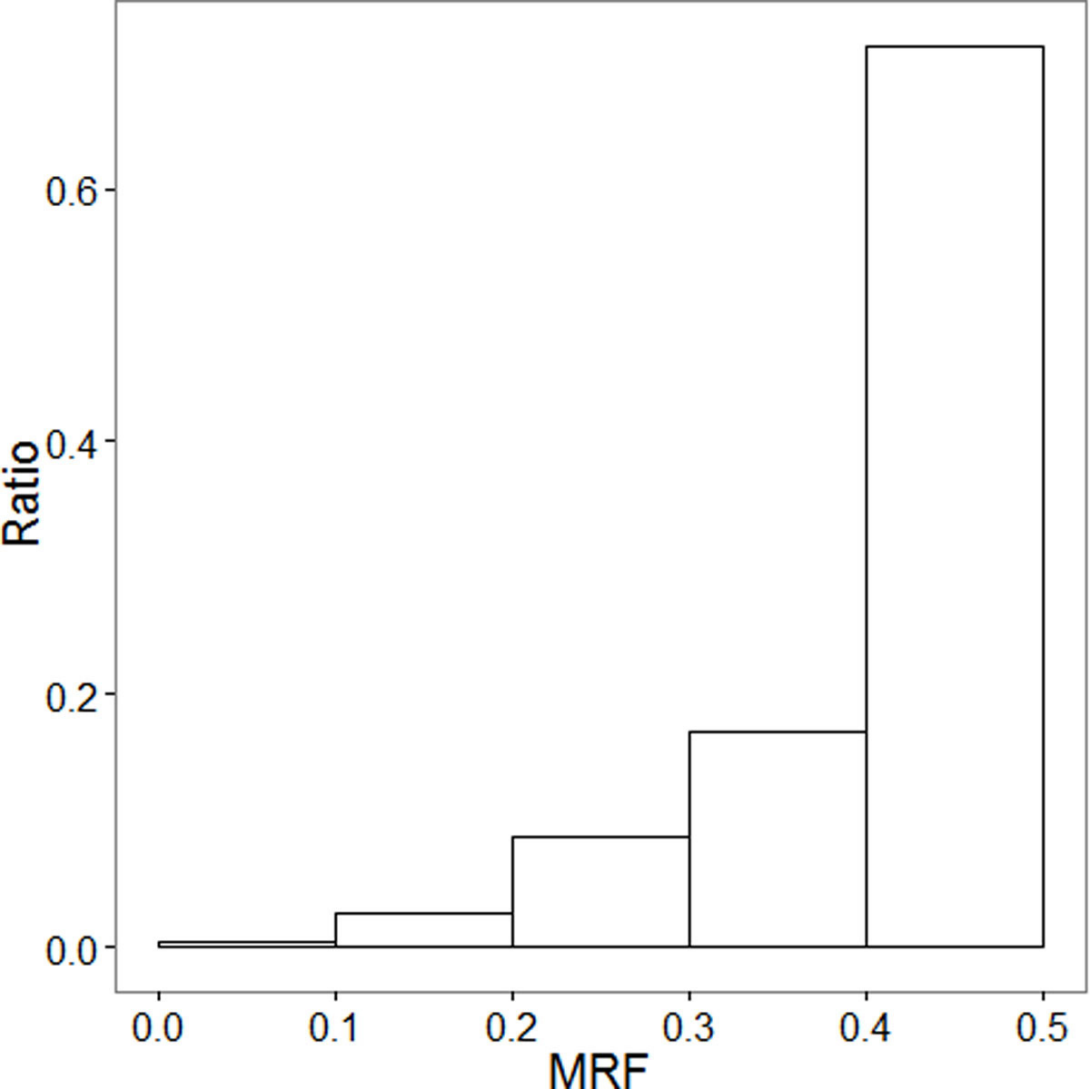
**Histogram of MRF for conflicting R(i, C)s**.

We calculated MRF values for 203,885 conflicting rule groups that have been filtered by four contexts (Figure 4). We found most of the conflicts are balanced. Out of 203,885 rule groups, 192,197 (94.3%) had MRF values larger than 0.25. On the contrary, only 11,688 (5.73%) rule groups had MRF values less than 0.25. Therefore, we conclude that there is insufficient evidence to eliminate one relation (a specific 'increase' or 'decrease' rule group) and it is necessary to consider more context types to resolve the conflicts; such contexts may include measurement time, ethnic origin or dependency between other entities.

We calculated MRF values for 310 conflicting rule groups in KEGG. Out of 310 rule groups, 283 (91.3%) were *balanced *conflicts. On the contrary, 27 (8.71%) were *dominating *conflicts. There are fewer conflict among rule groups from KEGG, and the most conflict are *balanced *conflict, too.

### Case study: *dominating *conflicts

We analyzed some of *dominating *conflicts showing very small MRF, and discovered that main reason of unbalance is due to text mining error.

For example, the following sentence is a sentence in PMID15799207 of PubMed; "Effective control of **growth hormone **should, with long-term use, **reduce **morbidity and mortality from acromegaly and has been shown to result in partial involution of the **pituitary adenoma **in the majority of treated patients." A rule was extracted, it had 'growth' which was tagged as a *LEFT-ENTITY *and 'pituitary adenoma' which was tagged as a *RIGHT-ENTITY*. This *rule *had not to be extracted, but it was extracted as a rule in *decrease rule subgroup*. This error occurred because 'reduce' between the two entities was tagged as a relation word. A true verb as a relation word of the two entities is 'has been shown', and wrongly tagged 'reduce' is used to mean that effective control of growth hormone reduces morbidity and mortality from acromegaly.

A sentence in PMID19759287 is "... a specific form of **neuronal death **induced by NMDAR overstimulation, dramatically **decreases **Kidins220/ARMS levels in cortical neurons and in a model of **cerebral ischemia**." A rule was extracted. It had 'neuronal death' which was tagged as a *LEFT-ENTITY *and 'cerebral ischemia' which was tagged as a *RIGHT-ENTITY*. This *rule *had not to be extracted, but it was extracted as a rule in *decrease rule subgroup*. This error occurred because 'decrease' between the entities was tagged as a relation. Here, 'decreased' is used to mean that exitotoxicity reduces Kidins220/ARMS.

### Case study: *balanced *conflicts

**Figure 5:**
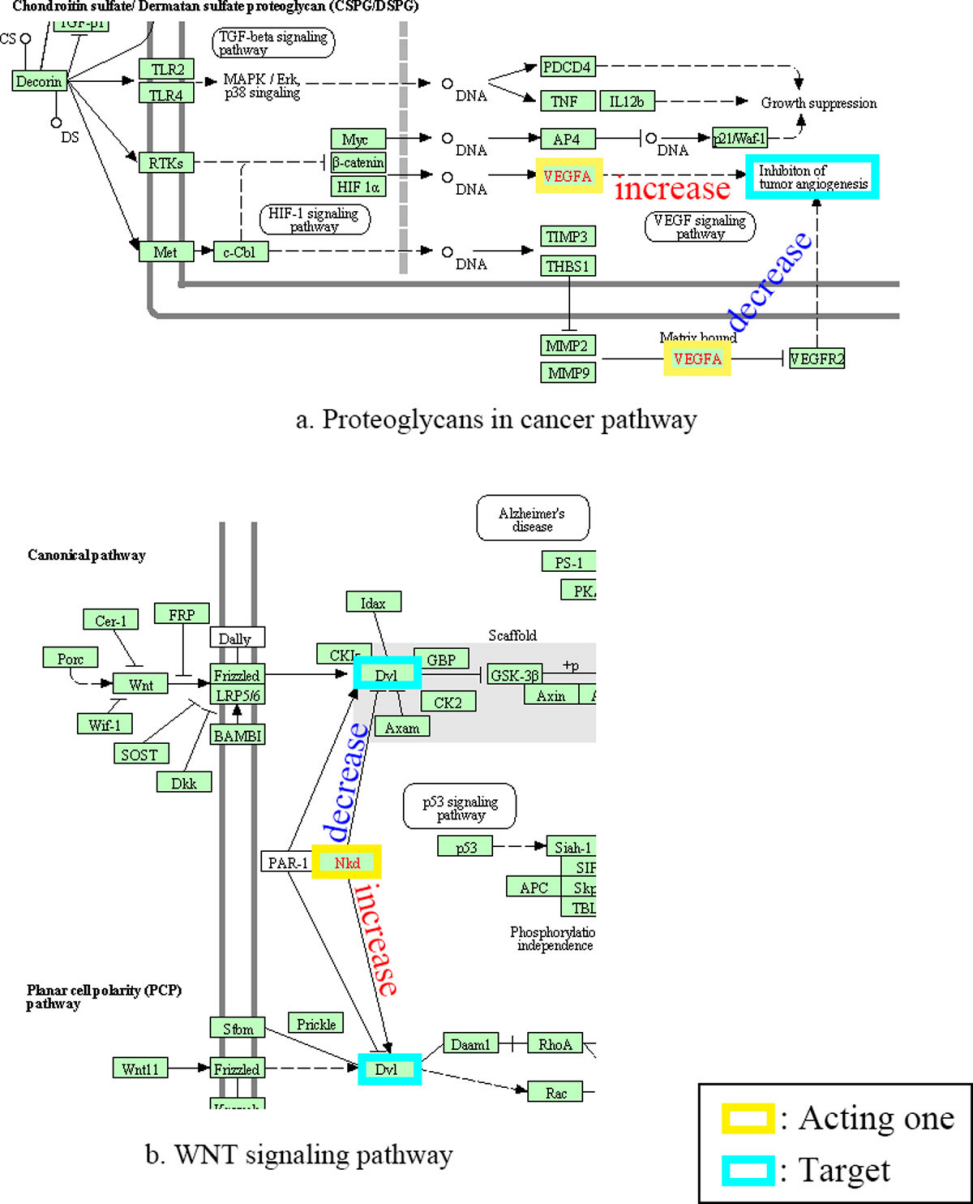
**Dual function of genes**. a show dual function of VEGFA in proteoglycans in cancer pathway, and b shows dual function of NKD1 in WNT pathway.

We analyzed some balanced conflicts. There are cases where difference of context which were not yet considered or informed, make conflict. Here are some examples. Vascular endothelial growth factor A (VEGFA) inhibits tumor angiogenesis. But it activates when bound to matrix [17]. This dual function is also shown in 'proteoglycans in cancer pathway' in KEGG (Figure 5a). Naked cuticle homolog 1 (NKD1) inhibits dishevelled segment polarity protein 1 (DV1) in canonical WNT signalling pathway. It, however, activates DV1 in Planar cell polarity (PCP) WNT pathway [18]. This is also in 'WNT signalling pathway' in KEGG (Figure 5b).

## Discussion

### Number of conflicts and resource quality

We measured and analyzed the conflicting rule groups from literature and a public database. As a result, conflicting rule groups occurred much more frequently in the literature than the public database, indicating that the public database are more reliable than literature mining. From this conclusion, we would be able to assign weights as reliability to *rules *based on its resource. Then, we could adjust the weights when we perform simulation or inference on constructed networks.

### Improving literature mining techniques

The measurement of conflicts among data extracted by literature mining can be used to identify literature mining errors. *Dominating *conflicts, which are heavily weighted on one side (either ***R***(***i***)^+ ^or ***R***(***i***)^-^), may be results of literature mining mistakes. Thus, through checking the weak rule groups, we could easily identify where literature mining process were incorrect. By finding and correcting these literature mining mistakes, literature mining techniques can be improved, resulting in more precise results.

### Biological meaning of *balanced *conflicts

Contrary to *dominating *conflicts, some conflicts have fairly even distribution on the both sides. We can consider these *balanced *conflicts as being due to dual functions rather than simple literature mining mistakes. Actually, some entities, even though they lie in the same context, have dual functions to the same object for the homeostasis and robustness of a biological process [19]. We expect that we could discover new dual functions by analyzing these *balanced *conflicts.

## Conclusion

In this study, we introduced a novel systematic analysis for identifying biological pathways with conflict information. The unexpectedly high rate of conflicts could be explained by two different ways: context specific dual functions and erroneous information. We provided a novel scoring measure, minor rule frequency (MRF), as a potential discriminatory feature for extracting novel dual-role information. By cleaning pathway information, we expect all subsequent analysis including network connectivity, dynamics and association based annotation to be improved and more useful in the current data-driven research era.

## Abbreviations

KEGG: Kyoto encyclopedia of genes and genomes; MC: manual curation; LM: literature mining; KGML: KEGG Markup Language; NER: Named Entity Recognition; OG: organ; CT: cell-type; DS: disease; DR: drug; MeSH: Medical Subject Heading; MRF: minor rule frequency; VEGFA: Vascular endothelial growth factor A; NKD1: Naked cuticle homolog 1; DV1: dishevelled segment polarity protein 1; PCP pathway: Planar cell polarity pathway

## Competing interests

The authors declare that they have no competing interests.

## Authors' contributions

DL and SK developed idea of this research and supported writing article. SY wrote article, extracted rule from literatures with KP and DJ, and make an algorithm to judge conflict of rules. JJ and HY wrote article, extracted rule from KEGG, and draw graphs. MK analyzed conflicting rules and draw graphs. SC searched previous works and draw graphs.

## Declarations

Publication costs for the article were sourced from Doheon Lee who is one of corresponding authors.

This article has been published as part of *BMC Medical Informatics and Decision Making *Volume 15 Supplement 1, 2015: Proceedings of the ACM Eighth International Workshop on Data and Text Mining in Biomedical Informatics. The full contents of the supplement are available online at http://www.biomedcentral.com/bmcmedinformdecismak/supplements/15/S1.

## References

[B1] Kamburov A Pentchev K Galicka H Wierling C Lehrach H Herwig R ConsensusPathDB: toward a more complete picture of cell biology Nucleic acids research 2011 39 suppl 1 D712 D717 21071422 10.1093/nar/gkq1156 PMC3013724

[B2] Cerami EG Gross BE Demir E Rodchenkov I Babur Ö Anwar N Schultz N Bader GD Sander C Pathway Commons, a web resource for biological pathway data Nucleic acids research 2011 39 suppl 1 D685 D690 21071392 10.1093/nar/gkq1039 PMC3013659

[B3] Stobbe MD Swertz MA Thiele I Rengaw T Van Kampen AH Moerland PD Consensus and conflict cards for metabolic pathway databases BMC systems biology 2013 7 1 50 10.1186/1752-0509-7-50 23803311 PMC3703255

[B4] Ajibade AA Wang HY Wang RF Cell type-specific function of TAK1 in innate immune signaling Trends in immunology 2013 34 7 307 316 10.1016/j.it.2013.03.007 23664135

[B5] Kanehisa M Goto S KEGG: kyoto encyclopedia of genes and genomes Nucleic acids research 2000 28 1 27 30 10.1093/nar/28.1.27 10592173 PMC102409

[B6] Ihaka R Gentleman R R: a language for data analysis and graphics Journal of computational and graphical statistics 1996 5 3 299 314

[B7] Nobata C Dobson PD Iqbal SA Mendes P Tsujii Ji Kell DB Ananiadou S Mining metabolites: extrt the yeast metabolome from the literature Metabolomics 2011 7 1 94 101 10.1007/s11306-010-0251-6 21687783 PMC3111869

[B8] Cheng D Knox C Young N Stothard P Damaraju S Wishart DS PolySearch: a web-based text mining system for extracting relationships between human diseases, genes, mutations, drugs and metabolites Nucleic acids research 2008 36 suppl 2 W399 W405 18487273 10.1093/nar/gkn296 PMC2447794

[B9] Tsuruoka Y Tateishi Y Kim J-D Ohta T McNaught J Ananiadou S Tsujii Ji Developing a robust part-of-speech tagger for biomedical text Advances in informatics 2005 Springer 382 392

[B10] Leaman R Gonzalez G BANNER: an executable survey of advances in biomedical named entity recognition Pacific Symposium on Biocomputing: 2008 2008 652 663 18229723

[B11] LingPipe 2.3.0 http://www.alias-i.com/lingpipe

[B12] NERsuite: A Named Entity Recognition toolkit http://nersuite.nlplab.org

[B13] Lipscomb CE Medical subject headings (MeSH) Bulletin of the Medical Library Association 2000 88 3 265 10928714 PMC35238

[B14] Wishart DS Knox C Guo AC Cheng D Shrivastava S Tzur D Gautam B Hassanali M DrugBank: a knowledgebase for drugs, drug actions and drug targets Nucleic acids research 2008 36 suppl 1 D901 D906 18048412 10.1093/nar/gkm958 PMC2238889

[B15] Shannon P Markiel A Ozier O Baliga NS Wang JT Ramage D Amin N Schwikowski B Ideker T Cytoscape: a software environment for integrated models of biomolecular interaction networks Genome research 2003 13 11 2498 2504 10.1101/gr.1239303 14597658 PMC403769

[B16] Yu X Lin J Zack DJ Qian J Identification of tissue-specific cis-regulatory modules based on interactions between transcription factors BMC bioinformatics 2007 8 1 437 10.1186/1471-2105-8-437 17996093 PMC2194798

[B17] Shibuya M Structure and dual function of vascular endothelial growth factor receptor-1 (Flt-1) The international journal of biochemistry & cell biology 2001 33 4 409 420 10.1016/S1357-2725(01)00026-7 11312109

[B18] Bangi E Wharton K Dual function of the Drosophila Alk1/Alk2 ortholog Saxophone shapes the Bmp activity gradient in the wing imaginal disc Development 2006 133 17 3295 3303 10.1242/dev.02513 16887821

[B19] Hart Y Antebi YE Mayo AE Friedman N Alon U Design principles of cell circuits with paradoxical components Proceedings of the National Academy of Sciences 2012 109 21 8346 8351 10.1073/pnas.1117475109 PMC3361385 22562798

